# *At*MYB93 is a novel negative regulator of lateral root development in Arabidopsis

**DOI:** 10.1111/nph.12879

**Published:** 2014-06-06

**Authors:** Daniel J Gibbs, Ute Voß, Susan A Harding, Jessica Fannon, Laura A Moody, Erika Yamada, Kamal Swarup, Candida Nibau, George W Bassel, Anushree Choudhary, Julien Lavenus, Susan J Bradshaw, Dov J Stekel, Malcolm J Bennett, Juliet C Coates

**Affiliations:** 1 School of Biosciences, University of BirminghamBirmingham, B15 2TT, UK; 2 Centre for Plant Integrative Biology, School of Biosciences, University of NottinghamNottingham, LE12 5RD, UK; 3 School of Biosciences, University of NottinghamNottingham, LE12 5RD, UK

**Keywords:** abscisic acid, ARABIDILLO, auxin, endodermis, lateral roots, MYB, negative regulation

## Abstract

- Plant root system plasticity is critical for survival in changing environmental conditions. One important aspect of root architecture is lateral root development, a complex process regulated by hormone, environmental and protein signalling pathways.
- Here we show, using molecular genetic approaches, that the MYB transcription factor *At*MYB93 is a novel negative regulator of lateral root development in Arabidopsis.
- We identify *At*MYB93 as an interaction partner of the lateral-root-promoting ARABIDILLO proteins. *Atmyb93* mutants have faster lateral root developmental progression and enhanced lateral root densities, while *At*MYB93-overexpressing lines display the opposite phenotype. *AtMYB93* is expressed strongly, specifically and transiently in the endodermal cells overlying early lateral root primordia and is additionally induced by auxin in the basal meristem of the primary root. Furthermore, *Atmyb93* mutant lateral root development is insensitive to auxin, indicating that *At*MYB93 is required for normal auxin responses during lateral root development.
- We propose that *At*MYB93 is part of a novel auxin-induced negative feedback loop stimulated in a select few endodermal cells early during lateral root development, ensuring that lateral roots only develop when absolutely required. Putative *At*MYB93 homologues are detected throughout flowering plants and represent promising targets for manipulating root systems in diverse crop species.

## Introduction

Plant rooting systems are fundamental for absorbing nutrients and water, anchoring the plant to its substrate, and responding to internal and external signals. As plants are sessile, plasticity of their root system is critical for survival. Plant root architecture requires complex regulation during development in response to hormones, signalling molecules and environmental changes.

The root systems of most vascular plants are formed by branching of lateral roots (LRs) from a primary root (PR) that first develops during embryogenesis. This process has been studied in great detail in several flowering plants, particularly Arabidopsis (Osmont *et al*., 2007; Nibau *et al*., 2008; De Smet, 2012). LRs initiate from a specialized cell layer in the PR, the pericycle. In Arabidopsis and most other dicots, LRs are formed only from pericycle cells overlying the developing xylem tissue (the xylem pole pericycle). LR development involves stimulation and dedifferentiation of pericycle founder cells, which increase in size, re-enter the cell cycle, and divide asymmetrically to give rise to a lateral root primordium (LRP), which then emerges through the outer layers of the PR (Celenza *et al*., 1995; Laskowski *et al*., 1995; Malamy & Benfey, 1997; Casimiro *et al*., 2003; Kurup *et al*., 2005; Peret *et al*., 2009a; Vermeer *et al*., 2014). The endodermis, the cell layer immediately overlying the pericycle, has recently been identified as a key regulator of LR developmental progression (Duan *et al*., 2013; Marhavy *et al*., 2013; Vermeer *et al*., 2014). Feedback from the endodermis to the pericycle is required for LR initiation (Marhavy *et al*., 2013; Vermeer *et al*., 2014). Moreover, the endodermis undergoes local remodelling and morphological changes during the very early stages of LR development, to accommodate the developing LRP (Vermeer *et al*., 2014), and also regulates later LR emergence events (Duan *et al*., 2013).

LR development and changes in root architecture are brought about through a combination of hormone signalling, environmental cues and hormone-independent protein activity (Osmont *et al*., 2007; Nibau *et al*., 2008; Tian *et al*., 2014). The key hormone in the development of LRs is auxin, which regulates all stages of LR development (Osmont *et al*., 2007; Nibau *et al*., 2008; Fukaki & Tasaka, 2009; Peret *et al*., 2009a). LR development is also affected by the majority of other plant hormones (Osmont *et al*., 2007; Xue & Zhang, 2007; Nibau *et al*., 2008; Sun *et al*., 2009; Kapulnik *et al*., 2011; Ruyter-Spira *et al*., 2011; Duan *et al*., 2013), and crosstalk between hormones occurs (Fukaki & Tasaka, 2009).

A diverse range of proteins and transcription factors integrate the signals controlling LR development, as do proteins that control LR development in an apparently hormone- and signal-independent manner, referred to as ‘intrinsic’ LR regulators (Malamy, 2005; Osmont *et al*., 2007; Hruz *et al*., 2008; Nibau *et al*., 2008). ARABIDILLO proteins are one example of putative intrinsic LR regulators: ARABIDILLO1 and ARABIDILLO2 act redundantly to promote LR development (Coates *et al*., 2006; Nibau *et al*., 2011).

In this paper, we show that a small, previously uncharacterized subfamily of R2R3 MYB (myeloblastosis) transcription factors interacts with ARABIDILLO1, and that at least two of the MYBs play a role during LR development. We show that one member of this subfamily, *At*MYB93, is expressed exclusively and transiently in roots in the endodermal cells overlying developing LRPs. Mutant and overexpression analyses demonstrate that *At*MYB93 functions as a negative regulator of LR development. Furthermore, we show that *AtMYB93* is induced by auxin, and that *Atmyb93* mutants are insensitive to auxin specifically with respect to LR development. We propose that *At*MYB93 is part of a novel negative feedback loop stimulated specifically in the endodermis upon LR initiation to ensure that LRs are formed only in the correct place.

## Materials and Methods

### PCR primers

All primers used are listed in Supporting Information Table S1.

### Yeast two-hybrid screening/assays

The ARABIDILLO1 ARMADILLO (ARM) domain (amino acids 378–767) was cloned into pGBKT7 and used to screen a seedling root primary cDNA library (Sorrell *et al*., 2003). *AtMYB93*, *AtMYB53*, *AtMYB75/PAP1* (*PAP1* = *PRODUCTION OF ANTHOCYANIN PIGMENT 1*) and *AtMYB91/AS1* (*AS1 = ASYMMETRIC LEAVES1*) cDNAs were PCR-amplified from whole-seedling total RNA and cloned into pGADT7. Constructs were co-transformed into yeast strain AH109 and tested for protein–protein interactions following the manufacturer's protocols (Takara Biosciences, Otsu, Japan). We could not test the reciprocal interaction, as *At*MYB92 autoactivates the yeast two-hybrid system.

### Sequence analysis, alignment and phylogeny

**Fig. 1:**
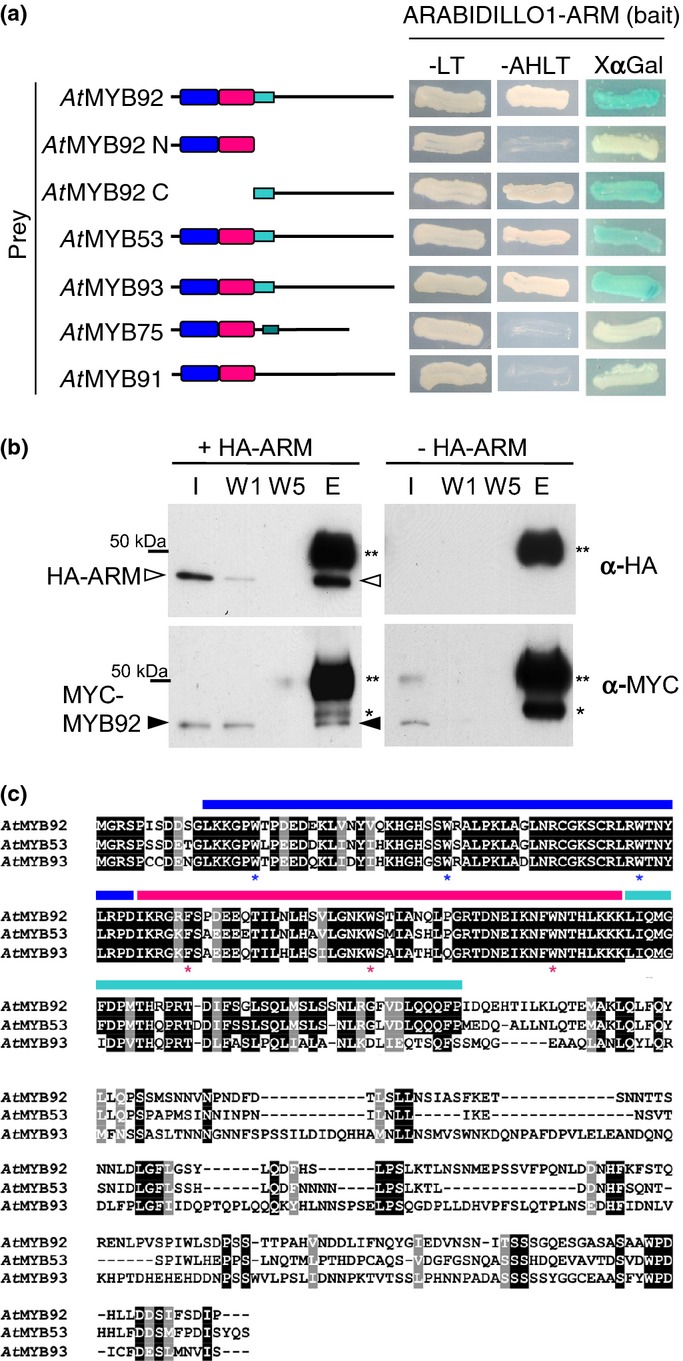
The ARABIDILLO-1 ARMADILLO (ARM) domain interacts with three related R2R3 MYB family proteins: *At*MYB92, -53 and -93. (a) Yeast two-hybrid interactions between the ARABIDILLO1 ARM-repeat domain and full-length Arabidopsis R2R3 MYB cDNAs expressed as GAL4-BD (GAL4-binding domain) and GAL4-AD (GAL4-activation domain) fusions, respectively. Growth on -LT (Leucine-Tryptophan) medium indicates successful co-transformation. Positive interactions are indicated by growth on -AHLT (Adenine-Histidine-Leucine-Tryptophan) medium and by blue colouration in the presence of X-α-gal. The ARABIDILLO-1 ARM-repeat domain interacts with *At*MYB92,*-*53 and -93, but not with more distantly related *At*MYB75/PAP1 (PAP1 = PRODUCTION OF ANTHOCYANIN PIGMENT 1) or *At*MYB91/AS1 (AS1 = ASYMMETRIC LEAVES1). The interaction is specific to the C-terminus downstream of the R2R3 MYB domain. Conserved R2 and R3 MYB domains are shown in blue and magenta; the cyan box denotes the conserved C-terminal motif of *At*MYB92, -53 and -93; the green box denotes a conserved C-terminal motif in *At*MYB75/PAP1. (b) Co-immunoprecipitation of N-terminally MYC-tagged *At*MYB92 (MYC-MYB92; closed arrowhead) with the N-terminally HA-tagged ARABIDILLO1 ARM domain (HA-ARM; open arrowhead). Proteins were synthesized *in vitro*, and co-incubated with anti-HA antibody. A control immunoprecipitation (IP) performed without the addition of HA-ARM was also conducted. I, input; W1/5, washes 1 and 5; E, elution; **, antibody heavy chain; *, nonspecific band in anti-MYC western blots. We performed similar experiments with *At*MYB93, but because of the size of *At*MYB93, it unfortunately could not be detected in the elution as it was occluded by the antibody heavy chain. (c) Alignment of the full-length amino acid sequences of *At*MYB92, *At*MYB53 and *At*MYB93. Black and grey shading denotes identical and similar amino acid residues, respectively. Blue and magenta bars denote conserved R2 and R3 MYB domains, respectively. The cyan bar denotes the conserved C-terminal motif unique to these three proteins. *, key conserved aromatic residues within the R2R3 MYB domain.

The initial MYB alignment (Fig. 1c) was conducted using ClustalX (Larkin *et al*., 2007) using the default settings. Alignments were annotated using Boxshade 3.21 (http://www.ch.embnet.org/software/BOX_doc.html), with the fraction of sequences that must agree for shading set at 1.0. For the phylogeny, putative full-length land plant *At*MYB93/92/53 homologues were identified using Blastp from fully sequenced land plant genomes via GenBank and Phytozome (Goodstein *et al*., 2012). Sequences were aligned using ClustalX and the alignment was refined manually in SeaView (Gouy *et al*., 2010). The phylogenetic tree was calculated using the maximum likelihood algorithm in SeaView on default settings, with 1000 bootstrap replicates. Similar trees were obtained using distance methods. The tree was displayed using TreeViewX (Page, 2002).

### Plant material and growth conditions

*Arabidopsis thaliana* (L.) Heynh Columbia (Col-0) ecotype was used. *Atmyb92* (SM_3_41690), *Atmyb93-1* (SALK_131752) and *Atmyb93-2* (GK-588A05) insertion lines were obtained from the JIC-SM, SALK and GABI-KAT collections, respectively (Tissier *et al*., 1999; Alonso *et al*., 2003; Kleinboelting *et al*., 2012). The *arabidillo1/2* double mutant was described previously (Coates *et al*., 2006), and was crossed with *Atmyb93-1* to yield a triple *arabidillo1/2/Atmyb93* mutant. Homozygous lines were identified by segregation analysis and by PCR/RT-PCR screening (Sessions *et al*., 2002).

Seeds were surface-sterilized using 20% Parozone™ (Jeyes, Cambridge, UK) and cold-treated (4°C) for 3 d before sowing. Seedlings were grown in sterile long-day conditions at 20–22°C on 0.5× Murashige and Skoog (MS) medium and 1% agar, pH 5.7, supplemented with hormones where required. Mature plants were grown in Levington M3 compost/vermiculite in the glasshouse (20–22°C, long days).

### Root assays

Seedlings were grown vertically. To calculate emerged LR density in different genotypes (see the Results section, Figs 4c, 8g, S2c, S3d, S7), visible emerged LRs were counted 7–12 d after germination under a compound microscope. For clarity, static data from one time-point are shown, but the same trends were seen over the entire time-course of each experiment. Root length was measured from digital photographs using ImageJ (http://rsb.info.nih.gov/ij/). The density of emerged LRs was defined as LRs per cm of PR for each seedling; similar trends were also observed when the ‘branching density’ (i.e. LR density per cm of PR branching zone; Dubrovsky & Forde, 2012) was calculated. For statistical analysis, the null hypothesis that there is no difference in mean LR density between wild-type and each mutant genotype was tested using pairwise *t*-tests. For LRP staging experiments, seedlings were cleared in Hoyer's medium and the number of LRPs at each developmental stage (Malamy & Benfey, 1997) was scored per root with a Leica DMRB microscope (Leica, Milton Keynes, UK); the percentage of LRPs at each developmental stage was then calculated for every root. For statistical analysis, the counts obtained are too low to apply a chi-squared test, so counts for each genotype were compared with those for the wild-type using a generalized likelihood test combined with a randomization procedure to generate *P*-values, in a manner analogous to methods for cDNA library comparison (Stekel *et al*., 2000; Herbert *et al*., 2008, 2011). For each strain comparison (wild-type versus mutant), the null hypothesis is that the frequency of LRPs at any given stage is the same between the two strains; the alternative hypothesis is that these frequencies are different. The log likelihood ratio of the observed frequencies under the two hypotheses was constructed using multinomial distributions to generate the test statistic. To generate a *P*-value, 10 000 simulated data sets were constructed using a multinomial distribution and the null hypothesis frequencies, and a test statistic was computed for each simulated data set. The *P*-value is approximated by the proportion of test statistics in the simulated data sets that are more extreme than the test statistic for the true data. Error bars were calculated using the standard error for a proportion, equal to sqrt(*p*(1 – *p*)/*n*), where *p* is the proportion and *n* is the population size.

For hormone treatments (see the Results section, Fig. 7a–d), seedlings were grown for up to 12 d on 0.5× MS plates containing indole-3-acetic acid (IAA), abscisic acid (ABA) and naphthylphtalamic acid (NPA) or relevant solvent control, and emerged LR density was calculated as above. For statistical analysis, the null hypothesis that there is no difference between the behaviour of wild-type and that of *Atmyb93* mutants under each treatment was tested using one-way analysis of variance (ANOVA) followed by a Tukey's multiple comparison test.

For LRP induction experiments to assess the rate of LRP development, *c*. 20 seedlings per genotype were grown on vertical plates for 3 d before rotating the plate 90° to induce formation of a single LRP. Seedlings were cleared in Hoyer's medium after either 18 or 42 h, and the stage of each induced LRP was scored at high magnification with a Leica DMRB microscope. For statistical analysis, a generalized likelihood test combined with a randomization procedure was applied as for the staging analysis as above.

### Cloning and construct generation for transgenic plants

The full-length *At*MYB93 promoter sequence (*c*. 1.6 kb upstream of the start codon) was amplified from Col-0 genomic DNA and cloned into pBI101 to make a *pAtMYB93::GUS* reporter. *35S::MYC-AtMYB93* and *35S::MYC-AtMYB93-YFP* were constructed in pGreen0229 (Hellens *et al*., 2000). Constructs in *Agrobacterium tumefaciens* strain GV3101 were transformed into Arabidopsis by floral dip (Clough & Bent, 1998). Protoplast transfection was carried out as described previously (Nibau *et al*., 2011).

### Promoter::GUS assays and imaging

Seedlings were assayed for β-glucuronidase activity according to standard protocols (Weigel & Glazebrook, 2002). Tissue was cleared through an ethanol/glycerol series and mounted in 50% glycerol for light microscopy. For hormone treatments, seedlings were grown vertically for 7 d before treatment with 1 μM IAA or 1 μM ABA in liquid 0.5× MS for 8 h and subsequent GUS staining. Sample preparation before confocal microscopy was carried out as described previously (Truernit *et al*., 2008). GUS/GFP imaging was carried out using Leica SP2 confocal microscopes.

### RT-PCR and qRT-PCR

To test mRNA induction by phytohormones, 8-d-old seedlings were treated for 8 h in liquid 0.5× MS supplemented with IAA or ABA. To examine gene expression during LRP progression, LRs were induced using a gravity stimulus. Total RNA was extracted using an RNeasy plant mini kit (Qiagen, Venlo, Netherlands) and cDNA prepared using Superscript II Reverse Transcriptase (Invitrogen, Carlsbad, CA, USA) with oligodT primers or random hexamer primers. qRT-PCR was carried out using the SensimixdT kit (Quantace, London, UK) or the SensiMix SYBR (2x) (Bioline, London, UK) using 50 ng of cDNA template per reaction (endogenous control: *ACTIN-2*). Primers were designed using PrimerExpress software (ABI, Waltham, MA, USA). All PCRs were carried out using an ABI Prism 7000 instrument (Applied Biosystems, Waltham, MA, USA) or the LightCycler® 480 (Roche, Basel, Switzerland) with default thermocycling conditions. qRT-PCR results were analysed using the comparative *C*_T_ method (Schmittgen & Livak, 2008). Three biological replicates were carried out, each containing three technical replicates.

To compare *AtMYB93* expression levels in wild-type and *Atmyb93-1*, RNA extraction, cDNA synthesis and qRT-PCR were carried out as above on Col-0 and *Atmyb93-1* root tissue. Two biological replicates were carried out, each containing four technical replicates. To present the data, the Col-0 expression level was set to 1.

### Co-immunoprecipitation (coIP) experiments

The ARABIDILLO1 ARM domain (in pGBKT7, which incorporates an N-terminal HA tag) and the full-length *At*MYB92 (in pGADT7, which incorporates an N-terminal MYC tag) were translated *in vitro* using the TNT® T7 Coupled Reticulocyte Lysate System (Promega, Madison, WI, USA). Translated proteins (or nonprotein controls) were mixed and incubated in immunoprecipitation (IP) buffer (Nibau *et al*., 2011) at 4°C with rotation. HA-ARM was immunoprecipitated using EZView™ Red Anti-HA Affinity Gel (Sigma-Aldrich) and detected by western blot using anti-HA. Co-immunoprecipitated MYC-*At*MYB92 was detected by anti-MYC western blot.

### Western blotting

Seven-day-old seedlings were ground in liquid nitrogen and mixed with protein extraction buffer (125 mM Tris-HCl, pH 8.8, 1% SDS, 10% glycerol and 50 mM Na_2_S_2_O_5_) supplemented with protease inhibitor cocktail (Roche). For MG132 treatments, seedlings were preincubated for 2 h with MG132 or a dimethyl sulfoxide (DMSO) control. Equal protein amounts were resolved by sodium dodecyl sulphate–polyacrylamide gel electrophoresis (SDS-PAGE) and transferred to PVDF using a Mini Trans-Blot electrophoretic transfer cell (Bio-Rad, Hercules, CA, USA). Membranes were probed with primary antibodies: anti-MYC (Santa Cruz, Dallas, TX, USA), 1 : 1000; anti-α-tubulin (Sigma), 1 : 5000. Horseradish peroxidase (HRP)-conjugated anti-mouse secondary antibody (Santa Cruz) was used at 1 : 10 000. Immunoblots were developed to film after using the ECL western blotting substrate (Pierce, Rockford, IL, USA).

## Results

### ARABIDILLO1 interacts with a specific group of related R2R3 MYB transcription factors

Previously we demonstrated that Arabidopsis ARABIDILLO proteins are positive regulators of LR initiation, containing an F-box, leucine-rich repeats and ARM repeats (Coates *et al*., 2006; Nibau *et al*., 2011). The full-length proteins are unstable, being turned over by the proteasome (Nibau *et al*., 2011). However, the ARABIDILLO1 ARM domain by itself is stable (Nibau *et al*., 2011), and ARM repeats are hypothesized to mediate protein–protein interactions (Coates, 2003). Thus, to understand the mechanism by which ARABIDILLO proteins function to promote LR development, we used the ARABIDILLO1 ARM domain as bait in a yeast two-hybrid screen of a seedling root cDNA library (Sorrell *et al*., 2003). We isolated a full-length clone of the R2R3 MYB transcription factor *At*MYB92 (At5g10280) as a putative interaction partner of ARABIDILLO1 (Fig. 1a). To specify the region(s) of the *At*MYB92 protein that interacts with the ARABIDILLO1 ARM domain, we tested truncated *At*MYB92 constructs in the two-hybrid system. The C-terminus of *At*MYB92 (*At*MYB92C; amino acids 113–334), downstream of the R2R3 DNA-binding domain, interacts with the ARABIDILLO1 ARM, while the N-terminus (*At*MYB92N; amino acids 1–112) does not (Fig. 1a). The interaction between the ARABIDILLO1 ARM and *At*MYB92 was confirmed by co-immunoprecipitation (Fig. 1b).

*At*MYB92 is one member of a small subfamily of Arabidopsis R2R3 MYB transcription factors, which also contains *At*MYB93 (At1g34670) and *At*MYB53 (At5g65230) (Kranz *et al*., 1998; Stracke *et al*., 2001). All three proteins share a conserved 41 amino acid motif downstream of the R2R3 MYB DNA-binding domain (26 amino acids longer than that identified by Kranz *et al*., 1998) that is not found in other Arabidopsis MYB proteins (Fig. 1c; Stracke *et al*., 2001; Dubos *et al*., 2010). Our alignments agree with previous phylogenetic analyses demonstrating that *At*MYB92 and *At*MYB53 are more similar to each other than either is to *At*MYB93 (Fig. 1c; Stracke *et al*., 2001). To test whether these related proteins also interact with ARABIDILLO1, we cloned full-length *AtMYB93* and *AtMYB53* cDNAs. Both proteins interact with the ARABIDILLO1 ARM domain, while the more distantly related R2R3 MYB proteins *At*MYB91 (AS1) and *At*MYB75 (PAP1) do not (Fig. 1a).

### The ARABIDILLO-interacting MYB subfamily is present only in flowering plants

**Fig. 2:**
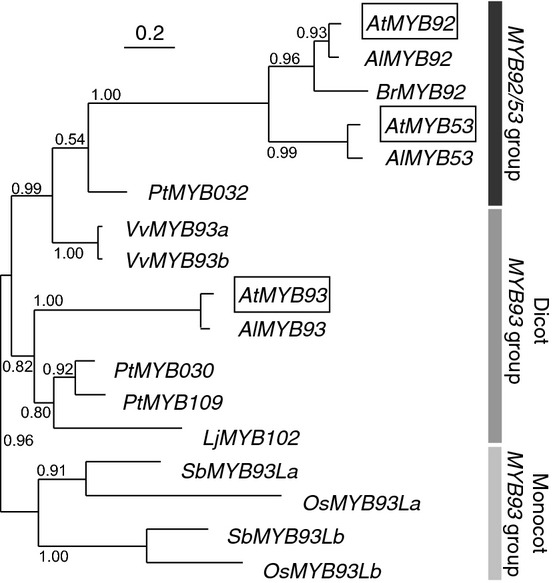
*AtMYB92*, *-53* and *-93* homologues are found in flowering plants. An inferred phylogenetic tree of full-length *At*MYB92, *-*53 and -93 protein homologues identified in the plant lineage from fully sequenced genomes is shown. Three main groups are identified: a group of dicot proteins most closely related to *At*MYB92/*At*MYB53 (black bar), a group of dicot proteins most closely related to *At*MYB93 (dark grey bar) and a group of monocot proteins similar to *At*MYB93 (light grey bar). The scale bar represents the average number of substitutions per column in the sequence alignment used for generating the phylogeny. Bootstrap values > 0.5 are shown. *At*, *Arabidopsis thaliana*; *Al, Arabidopsis lyrata*; *Br*, *Brassica rapa*; *Pt*, *Populus trichocarpa*; *Vv*, *Vitis vinifera*; *Lj*, *Lotus japonicus*; *Sb*, *Sorghum bicolor*; *Os*, *Oryza sativa*.

As ARABIDILLO proteins are present in all land plants, including those without LRs (Nibau *et al*., 2011; Moody *et al*., 2012), we searched for putative ARABIDILLO-interacting MYB homologues throughout the land plant lineage. We used the C-terminal regions of *At*MYB93, *At*MYB92 and *At*MYB53 in Blastp searches to identify full-length proteins in other species with similarity to the three ARABIDILLO-interacting MYBs across the entire length of their C-terminus. Putative homologues are present in both dicot and monocot flowering plants, which possess LRs, but are apparently absent from bryophytes (which lack true multicellular roots) and lycophytes (which lack LRs) (Fig. 2; alignment in Fig. S1). Thus, unlike ARABIDILLO proteins, which are found throughout the plant kingdom, putative homologues of ARABIDILLO-interacting MYB proteins are found only in flowering plants, which have multicellular, branched root systems that generate LRs.

### *AtMYB93* has root-specific expression and is up-regulated during LR development

**Fig. 3:**
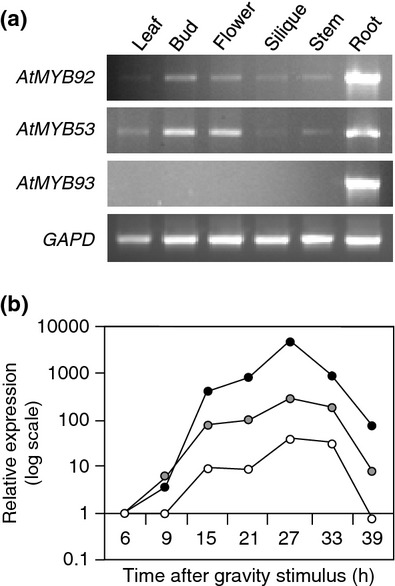
Arabidopsis *AtMYB92*, *-93* and *-53* expression is enriched in roots and induced in developing lateral root primordia (LRP). (a) Tissue-specific expression of *AtMYB92*, *-53* and *-93*. *AtMYB93* mRNA is detected exclusively in roots, while *AtMYB92* and AtMYB53 mRNAs both show root-enriched expression. Data are representative of more than three biological replicates. (b) Relative *AtMYB92* (white circles), *-53* (grey circles) and *-93* (black circles) gene expression over time in developing LRP (shown as log scale). mRNA abundance was analysed by qRT-PCR at the times indicated following initiation of LRPs with a gravity stimulus. All three genes are up-regulated during early stages of LR development, and are down-regulated again at later time-points. This induction was particularly strong for *AtMYB93* (> 8000-fold increase by 27 h).

To determine where *AtMYB92*, *-93* and *-53* genes are active, we examined their mRNA abundance in different plant tissues by semiquantitative RT-PCR (Fig. 3a). *AtMYB92* and *AtMYB53* mRNAs were detected throughout the plant but were enriched in roots, whereas *AtMYB93* cDNA was amplified only from roots, indicating that its expression is restricted to this tissue (Fig. 3a).

Next we investigated whether these MYBs might play a specific role in LR development by examining their temporal expression by qRT-PCR in developing LRs that had been artificially induced by a gravitropic (bending) stimulus (Lucas *et al*., 2008; Peret *et al*., 2012). We detected an early increase in the mRNA levels of all three MYBs in ‘bend’ sections (excised from the PR) that were forming LRs (Fig. 3b), between 6 and 27 h after the gravitropic stimulus (from the first cell division to stage III in wild-type roots) (Peret *et al*., 2012). Following this initial up-regulation, levels decreased at later time-points (Fig. 3b). This increase was particularly striking for *AtMYB93*, which was up-regulated several thousand-fold, while the increases observed for *AtMYB92* and *AtMYB53* expression were > 100- and 10-fold lower, respectively (Fig. 3b). The root-restricted localization of *AtMYB93* expression, coupled with its very strong induction at sites where LRs are forming, suggested that *At*MYB93 might function specifically during LR development.

### *At*MYB93 is a negative regulator of lateral root development

**Fig. 4:**
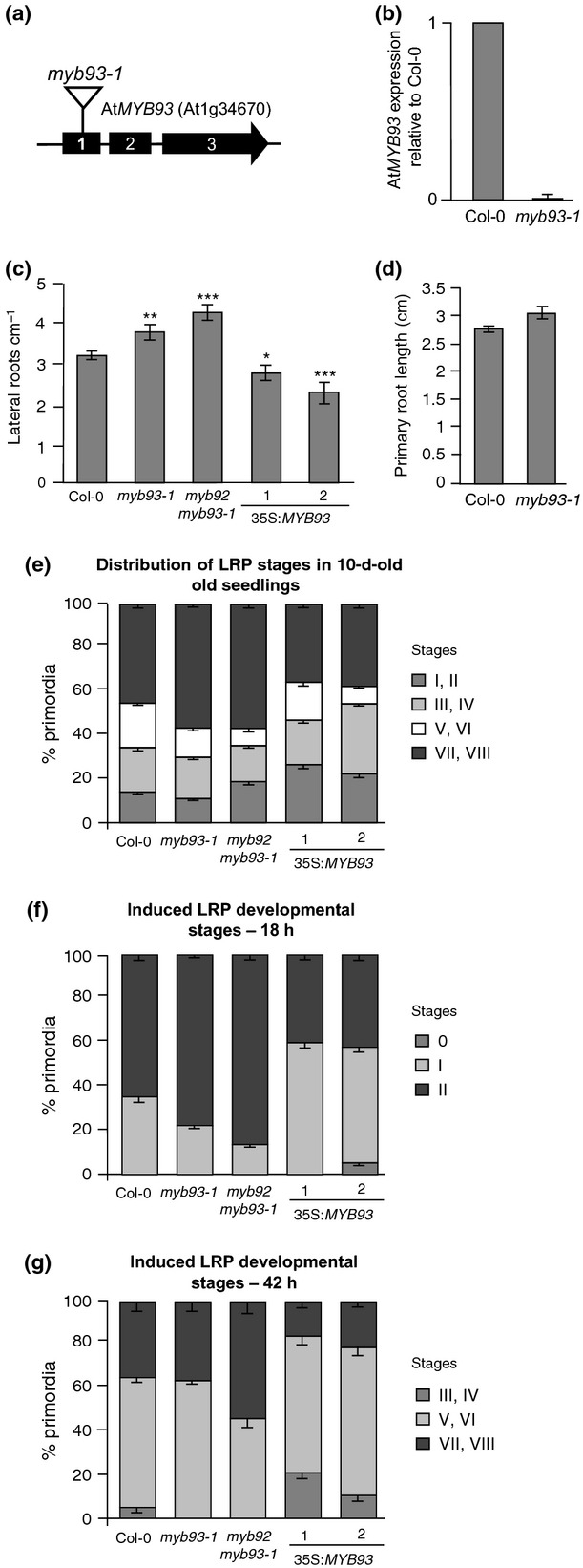
Arabidopsis *At*MYB93 is a negative regulator of lateral root development. (a) Intron–exon structure of the *AtMYB93* gene coding region, showing the location of the *Atmyb93-1* T-DNA insertion in exon 1. (b) qRT-PCR of *AtMYB9*3 transcript in wild-type and *Atmyb93-1* seedlings showing absence of *AtMYB93* expression in the mutant relative to Columbia (Col-0). (c) Emerged lateral root (LR) densities in 10-d-old seedlings of *MYB* mutant and overexpressing lines. The *Atmyb93-1* and *Atmyb92/Atmyb93-1* mutants have a greater LR density than wild-type plants. Two independent *35S::MYC::AtMYB93* lines have reduced LR density compared with wild-type plants. Data are from 10-d-old seedlings (*n* > 60 for all lines). (d) Primary root (PR) length is not significantly altered in *Atmyb93-1* mutants compared with wild-type plants (*n* > 60). (e) Distribution of lateral root primordia (LRPs) at different developmental stages in 10-d-old seedlings of wild-type, *Atmyb93-1* and *Atmyb92/93-1* mutants and *At*MYB93-overexpressing lines. *Atmyb93-1* and *Atmyb92/93-1* mutants have a larger proportion of LRPs at later stages (VII/VIII), while *At*MYB93-overexpressing lines have a larger proportion of LRPs at earlier stages (I–IV), indicating differences in the rate of LR development in the different mutant lines. *P* < 0.05 for wild-type versus 35S:*At*MYB93 line 2 using a generalized likelihood test combined with a randomization procedure as described in the Materials and Methods section. (f, g) Percentage of LRPs at specific developmental stages 18 h (f) and 42 h (g) after induction of a single LRP per seedling using a gravity stimulus. *Atmyb93-1* single and *Atmyb92/Atmyb93-1* double mutants have faster LR initiation and LRP progression, as indicated by a larger proportion of induced primordia at later stages relative to wild-type, whereas *35S::MYC::AtMYB93* overexpression lines have slower LRP initiation and progression, as indicated by a larger proportion of primordia at earlier stages relative to wild-type (*n* > 20). All genotypes show a significant (*P* < 0.05) difference in distribution at 42 h using a generalized likelihood test combined with a randomization procedure as described in the Materials and Methods. Error bars (c, d), standard error of the mean. *t*-tests: *, *P* < 0.05; **, *P* < 0.01; ***, *P* < 0.001. Error bars (e–g), standard error of proportion.

As *AtMYB93* is the ARABIDILLO-interacting MYB most strongly and specifically expressed during LR development, we isolated a homozygous *Atmyb93* T-DNA insertion mutant, *Atmyb93-1*, in which no *AtMYB93* expression could be detected (Fig. 4a,b). When root growth and LR development were examined in detail, we found that the LR density (emerged LRs per cm of PR) was significantly higher in the *Atmyb93-1* mutant than in wild-type plants (Fig. 4c). Emerged LR density was calculated for the entire length of the PR, for which no significant differences were observed between the genotypes (Fig. 4d). A second independent allele, *Atmyb93-2*, also showed a similar increase in emerged LR density (Fig. S2). We also obtained a homozygous T-DNA insertion in the *AtMYB92* gene, but this mutant showed identical PR and LR morphology to wild-type (Fig. S3), suggesting either that *AtMYB92* has no root function (possibly because of its much lower expression at sites of LR induction; Fig. 3b) or that it functions redundantly with *AtMYB93*. To test the latter possibility, we constructed an *Atmyb92/Atmyb93-1* double mutant. This mutant showed a slightly, but significantly, stronger phenotype than the single *Atmyb93-1* mutant (*P* < 0.05 by ANOVA and Tukey's multiple comparisons test; Fig. 4c), implying some (but not complete) overlap of function. We were unable to analyse a triple *Atmyb* mutant as no insertion lines for *AtMYB53* are available. Next we generated transgenic Arabidopsis lines ectopically expressing MYC-tagged *At*MYB93 driven by the cauliflower mosaic virus 35S (CaMV35S) promoter (*35S::MYC-AtMYB93*) and confirmed overexpression by immunoblotting (Fig. 8d). These *AtMYB93*-overexpressing lines displayed the opposite phenotype to the *Atmyb93* loss-of-function mutants, namely a significantly reduced emerged LR density (Figs 4c, S2), and they also had a slight decrease in PR length (Fig. S4).

To further understand these altered LR densities in the mutants and overexpressing lines, we carried out a detailed LRP staging analysis for each genotype, examining the distribution of LRPs at each developmental stage along the full length of the root. Although there was no obvious build-up of LRPs at any particular stage that would indicate a major defect in the LRP emergence process, we found that the proportion of LRPs at late stages (VII and VIII (emerged)) was slightly higher in the *Atmyb93* and *Atmyb92myb93* mutants than in Col-0 (Fig. 4e). Moreover, the proportion of LRPs at early stages (I–IV) was greater in the *At*MYB93-overexpressing lines than in Col-0 (Fig. 4e). These data suggested that there might be differences in the rate of LRP progression through development in the mutants and overexpressing lines, which would account for the differences in emerged LR densities.

To test this possibility further, we used a gravitropic stimulus to induce formation of a single LRP in multiple seedlings of each genotype (Lucas *et al*., 2008; Peret *et al*., 2012). The developmental stage of each induced LRP was then recorded within these seedling populations at both 18 and 42 h after applying the stimulus. We found that LRPs in the *Atmyb93-1* and *Atmyb92/93-1* mutants progress through development faster than in wild-type, as indicated by a greater proportion of induced LRPs at later stages at both 18 and 42 h post-stimulus (Fig. 4f). By contrast, the speed of LRP progression in *35S::MYC-AtMYB93* seedlings was slightly delayed compared with wild-type, as the distribution of induced LRPs at both 18 and 42 h was shifted towards earlier stages compared with Col-0 (Fig. 4g).

Collectively, the increased speed of LR initiation and progression and emerged LR density of the *Atmyb93* mutants, coupled with the opposite phenotypes of overexpression lines, indicate that *At*MYB93 is a negative regulator of LR development.

### *AtMYB93* is specifically expressed in the endodermis at sites of early lateral root development

**Fig. 5:**
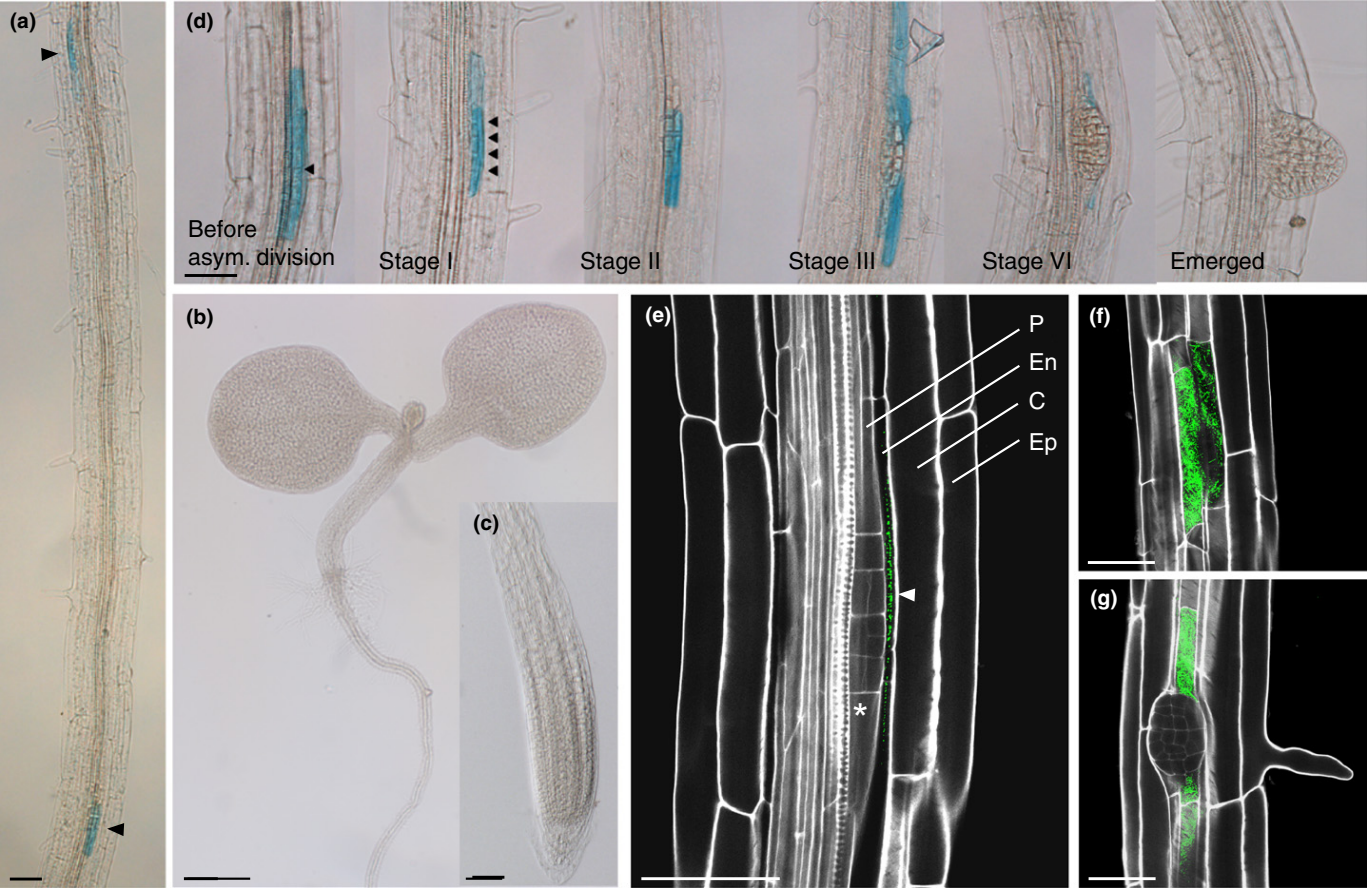
*AtMYB93* promoter activity is restricted to endodermal cells overlying early-stage lateral root primordia in Arabidopsis. (a) *pAtMYB93::GUS* expression in the mature root is restricted to sites of developing lateral root primordia (LRPs) (arrowheads). (b) *pAtMYB93::GUS* expression is absent from the cotyledons and hypocotyls of seedlings. (c) *pAtMYB93::GUS* expression is absent from the primary root (PR) tip. (d) Detailed expression analysis of *pAtMYB93::GUS* in developing LRPs. GUS activity in the endodermis is detected before the first asymmetric division, and throughout early stages (I–V) of development. During later stages (VI–VII), GUS activity fades and is completely absent upon emergence. Arrowheads show early cell divisions. (e) Confocal image of an early-stage LRP (asterisk) showing that *pAtMYB93::GUS* (green) is expressed exclusively in the endodermal layer overlying the primordia (arrowhead). P, pericycle; En, endodermis; C, cortex; Ep, epidermis. Cell walls are stained with propidium iodide. (f, g) Confocal images of LRPs showing that *pAtMYB93::GUS* expression is localized to the cells surrounding primordia as they develop through later stages. Cell walls are stained with propidium iodide. Bars: (a, c–e) 50 μm; (b) 200 μm.

To examine the timing and localization of *AtMYB93* expression in the root in more detail, we generated transgenic Arabidopsis plants expressing an *AtMYB93* reporter gene, consisting of the *AtMYB93* upstream region fused to a β*-glucuronidase* (*GUS*) reporter (*pAtMYB93::GUS*). In 3–7-d-old seedlings, *pAtMYB93* promoter activity was confined to regions of the root where LRs are forming (Fig. 5a), and was absent throughout the rest of the seedling (Fig. 5b,c). *pAtMYB93::GUS* expression was only detected early during LR development, initially before the first asymmetric cell divisions and then during the early stages of development (stages 0–IV; Fig. 5d). Expression faded during the later stages (V–VII) and was completely absent once the LR had emerged (Fig. 5d). The temporal activity of the *pAtMYB93* promoter correlated well with the *AtMYB93* mRNA expression profile observed during LRP progression (Fig. 3b), suggesting that the 1.6-kb promoter fragment used contains all of the regulatory elements needed for correct gene expression. Interestingly, *pAtMYB93* appeared to be only active in the cells overlying developing primordia, rather than within the LRPs themselves (Fig. 5d). To confirm this, we examined the cell-type-specific localization of *pAtMYB93* activity by confocal microscopy, revealing that the promoter is active exclusively in the endodermal cells that overlie early LRPs, and that surround later stage LRPs as they begin to emerge through the cortical layer of the root (Fig. 5e–g). This indicates that *At*MYB93 exerts its negative regulatory effect on LRP development from the endodermis, which has recently been identified as a critical tissue exerting feedback on the LR initiation and emergence process (Marhavy *et al*., 2013; Vermeer *et al*., 2014).

### *AtMYB93* gene expression is up-regulated by auxin specifically in the root basal meristem

**Fig. 6:**
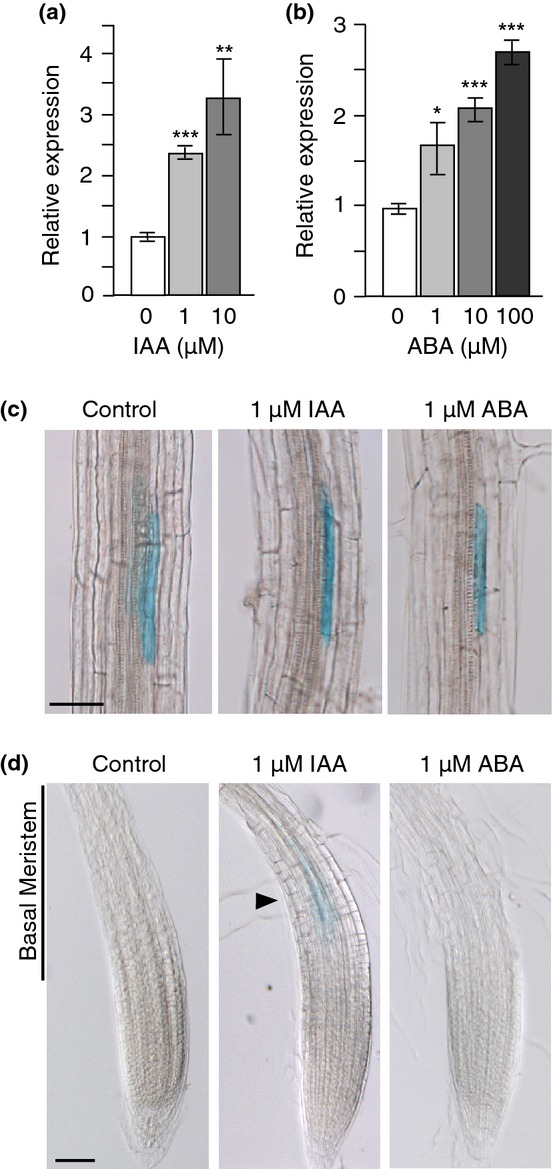
Arabidopsis *AtMYB93* expression is induced by auxin and abscisic acid (ABA). (a) qRT-PCR analysis of *AtMYB93* relative expression in 8-d-old seedlings treated with increasing concentrations of exogenous indole-3-acetic acid (IAA) (*n* = 3). (b) qRT-PCR analysis of *AtMYB93* relative expression in 8-d-old seedlings treated with increasing concentrations of exogenous ABA (*n* = 3). (c) *pAtMYB93::GUS* expression in lateral root (LR) primordia is not significantly altered in response to exogenous IAA or ABA treatment. (d) *pAtMYB93::GUS* expression is specifically induced in the basal meristem of the primary root (PR) in response to exogenous IAA (arrowhead). No *pAtMYB93::GUS* activity is observed in control or ABA-treated PRs. Seven-day-old seedlings are shown. Error bars, ± SE. *t*-tests: *, *P* < 0.05; **, *P* < 0.01; ***, *P* < 0.001. Bar: 50 μm.

LR development is regulated by many phytohormones (Nibau *et al*., 2008), and previous large-scale experiments have suggested that *AtMYB93* is regulated specifically by both auxin and abscisic acid (ABA) (Kranz *et al*., 1998; Vanneste *et al*., 2005; Yanhui *et al*., 2006; Winter *et al*., 2007; Lewis *et al*., 2013). To further our understanding of *AtMYB93* gene regulation, we analysed the effects of both of these phytohormones on *AtMYB93* gene expression in 8-d-old seedlings, using qRT-PCR. *AtMYB93* gene expression was up-regulated (*c*. 2- to 3-fold) by both auxin (IAA) and ABA in a dose-dependent manner (Fig. 6a,b) but not by other hormones (gibberellin (GA), salicylic acid (SA) and jasmonic acid (JA)), corroborating previous findings ((Kranz *et al*., 1998; Yanhui *et al*., 2006; Winter *et al*., 2007) and data not shown). This is in contrast to both *AtMYB92*, which is not induced by IAA or ABA, and *AtMYB53*, which is induced by ABA but not IAA (Fig. S5; Kranz *et al*., 1998; Vanneste *et al*., 2005; Yanhui *et al*., 2006; Winter *et al*., 2007; Lewis *et al*., 2013).

To validate the qRT-PCR data, and localize where *AtMYB93* induction was occurring in the root, we analysed *AtMYB93* promoter activity in response to auxin and ABA using *pAtMYB93::GUS*. Hormone application did not lead to significant temporal or spatial changes in the expression of *pAtMYB93::GUS* in the endodermal cells overlying LRPs (Fig. 6c). However, upon exogenous auxin application, additional weak *pAtMYB93::GUS* expression was detected specifically in the basal meristem of the PR (Fig. 6d), a region instrumental in determining the position and spacing of LRPs (De Smet *et al*., 2007). In response to ABA, no change in the intensity or pattern of *pAtMYB93::GUS* was seen either in the basal meristem (Fig. 6d) or in the rest of the plant. This suggests a different mode of regulation for *AtMYB93* gene expression in response to auxin compared with ABA. The spatial specificity of *AtMYB93*'s auxin induction suggests that the inhibitory role of *At*MYB93 during LR initiation is linked to auxin signalling, and may occur very early during LRP/pericycle cell priming in the basal meristem (De Smet *et al*., 2007).

### *Atmyb93* mutants show reduced sensitivity to auxin during lateral root development

**Fig. 7:**
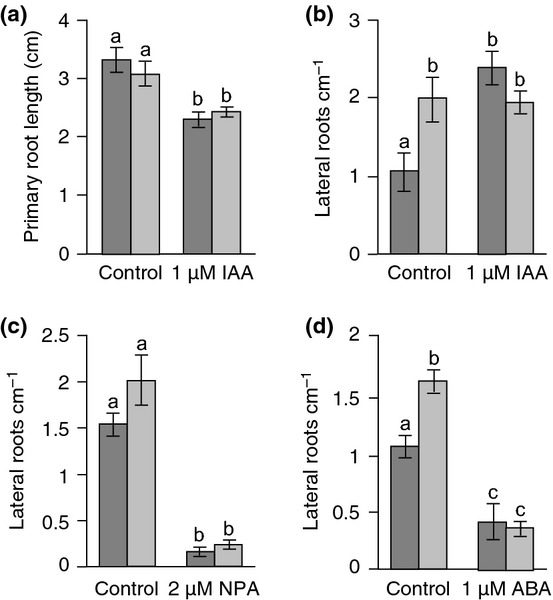
Arabidopsis *Atmyb93-1* mutants have reduced sensitivity to auxin for lateral root (LR) development. (a) *Atmyb93-1* primary root (PR) length is reduced by exogenous indole-3-acetic acid (IAA) similarly to wild-type. Nine-day-old seedlings are shown (*n* > 60). Error bars, ± SE. Different letters indicate means that differ significantly (*P* < 0.0001). Columbia (Col-0), dark grey bars; *Atmyb93-1*, light grey bars. (b) Exogenous IAA leads to an increase in total LR density in wild-type seedlings, but not in *Atmyb93-1* seedlings. Nine-day-old seedlings are shown (*n* > 60). Error bars, ±SE. Different letters indicate means that differ significantly (*P* < 0.05). (c) *Atmyb93-1* LR formation is blocked by naphthylphtalamic acid (NPA) similarly to wild-type. Eleven-day-old seedlings are shown (*n* > 60). Error bars, ±SE. Different letters indicate means that differ significantly (*P* < 0.0001). (d) *Atmyb93-1* emerged LR density is reduced by abscisic acid (ABA) similarly to wild-type. Eleven-day-old seedlings are shown (*n* > 60). Error bars, ±SE. Different letters indicate means that differ significantly (*P* < 0.0001).

As *AtMYB93* is an LR inhibitor up-regulated by both auxin and ABA, we tested the sensitivity of *Atmyb93-1* mutant LR development to both hormones. The concentrations of auxin (IAA) tested inhibit PR elongation and promote the initiation stage of LR development (Blakely *et al*., 1988; Laskowski *et al*., 1995; Coates *et al*., 2006; Ivanchenko *et al*., 2010). *Atmyb93-1* mutants responded to IAA similarly to wild-type with respect to PR elongation (Fig. 7a), but showed insensitivity to LR induction by exogenous IAA when applied at a concentration of 1 μM (Fig. 7b). No significant insensitivity of *Atmyb93-1* mutants was seen when using lower concentrations of auxin (25–500 nM) (data not shown).

As LR initiation and emergence both require auxin transport (Reed *et al*., 1998; Bhalerao *et al*., 2002), we tested the response of *Atmyb93-1* to the auxin transport inhibitor NPA. NPA inhibited LR development in the *Atmyb93-1* mutant similarly to in wild-type plants (Fig. 7c). Moreover, *Atmyb93-1* seedlings treated with NPA and then transferred to normal growth medium still formed more LRs than similarly treated wild-type seedlings (Fig. S6). This suggests that *AtMYB93* does not repress LR development via auxin transport pathways, but instead negatively affects auxin signalling. ABA inhibits LR emergence after the initiation stage, at concentrations (< 1 μM) that do not affect PR growth (De Smet *et al*., 2003). *Atmyb93-1* mutants responded as wild-type to ABA, showing a marked decrease in the number of emerged LRs present upon ABA treatment (Fig. 7d).

Thus, *Atmyb93-1* mutants are somewhat insensitive to auxin, specifically with respect to LR development, but show normal responses to auxin transport inhibitors and ABA. Collectively, these data suggest that *At*MYB93 is an LR-specific modulator required for normal auxin-signalling responses during LR development, and therefore represents a novel auxin-induced negative regulator of LR development.

### *At*MYB93 is not a degradation target of ARABIDILLOs

We identified *At*MYB93 as an interaction partner of the ARABIDILLO1 ARM domain in yeast. ARABIDILLO1 is an F-box protein proposed to facilitate ubiquitination and degradation of target protein partners, and *arabidillo1/2* double mutants have reduced LR densities (Nibau *et al*., 2011). Given the opposite phenotypes of *arabidillo* and *Atmyb93* loss-of-function mutants, one hypothesis is that ARABIDILLO proteins target *At*MYB93 for degradation.

**Fig. 8:**
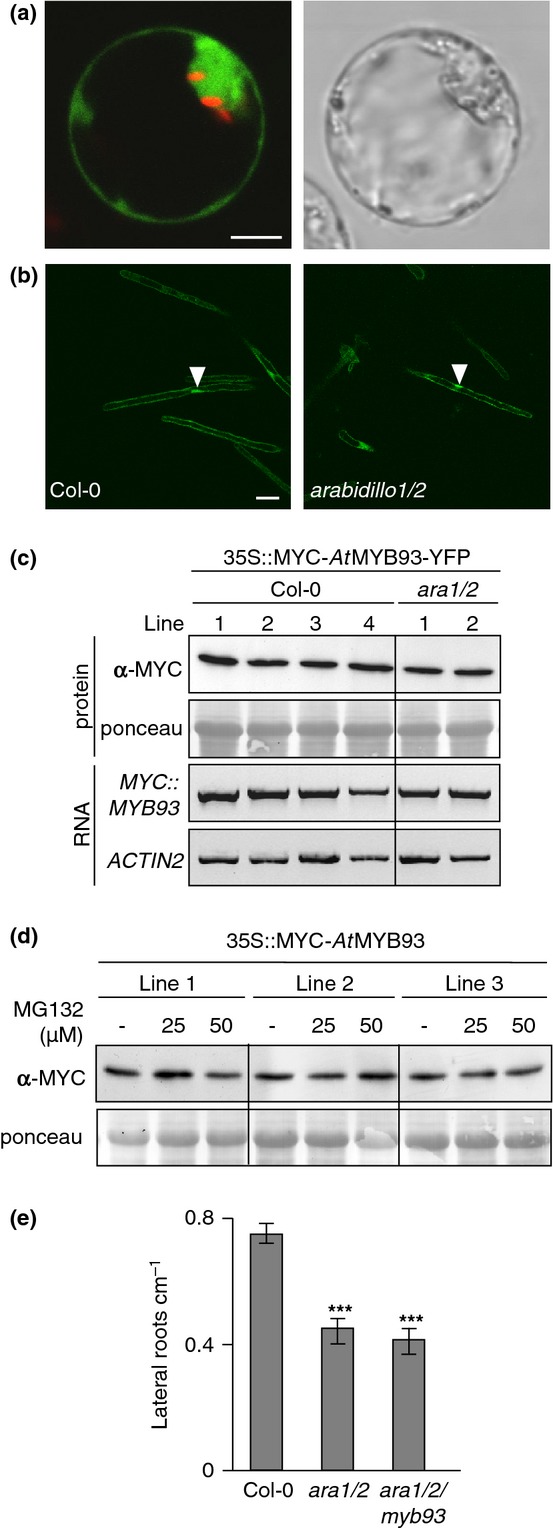
Arabidopsis *At*MYB93 does not appear to be a degradation target of ARABIDILLOs. (a) Fluorescent and brightfield confocal sections of an Arabidopsis protoplast showing nuclear and cytosolic localization of a 35S::MYC-*At*MYB93-YFP translational fusion protein. Bar, 10 μm. (b) Fluorescent confocal sections of root hairs of wild-type (left) and *arabidillo1/2* (right) seedlings stably expressing a 35S::MYC-*At*MYB93-YFP fusion protein, showing nuclear and cytosolic localization and equal protein intensities. Bar, 25 μm. (c) Western blot and RT-PCR analysis of MYC-*At*MYB93-YFP in wild-type and *arabidillo1/2* seedlings. There are no significant differences in expression levels and protein stability in the two genetic backgrounds, indicating that ARABIDILLOs do not regulate *At*MYB93 stability. (d) Western blot analysis of MYC-*At*MYB93 protein in seedlings treated with proteasome inhibitor MG132. MYC-*At*MYB93 stability is not enhanced in the presence of MG132, suggesting that *At*MYB93 is not regulated by the proteasome. (e) A triple *arabidillo1/arabidillo2/Atmyb93* mutant has a phenotype resembling that of the *arabidillo1/2* mutant (reduced emerged lateral root density). Error bars, ± SE. *t*-test: ***, *P* < 0.001.

To test this possibility, we generated a MYC-tagged *At*MYB93-YFP fusion protein driven from the CaMV35S promoter, which localized to both the nucleus and the cytosol of Arabidopsis protoplasts and stably transformed wild-type and *arabidillo1/2* mutant seedlings (Fig. 8a,b). The relative abundance of MYC-*At*MYB93-YFP was not enhanced in the *arabidillo1/2* mutant, suggesting that ARABIDILLOs do not regulate *At*MYB93 stability (Fig. 8b). To confirm this, we compared the protein levels of 35S::MYC-*At*MYB93-YFP in wild-type and *arabidillo1/2* mutant seedlings by western blotting and detected no difference in protein abundance (Fig. 8c). Moreover, treatment of transgenic 35S::MYC-*At*MYB93 seedlings with the proteasome inhibitor MG132 did not lead to an accumulation of the MYC-*At*MYB93 protein (Fig. 8d), suggesting that global *At*MYB93 stability is regulated neither by ARABIDILLOs nor by general proteasomal turnover. Furthermore, there was no change in *AtMYB93* mRNA levels in an *arabidillo1/2* background, and no change in *arabidillo* mRNA levels in an *Atmyb93-1* mutant background (Fig. S7), implying that the *AtMYB93* and *ARABIDILLO* genes do not affect each other's transcription. This suggests that the *At*MYB93–ARABIDILLO interaction may control LR development via a nonproteasomal mechanism. To test this suggestion genetically, we generated an *arabidillo1/arabidillo2/Atmyb93-1* triple mutant, which showed reduced LR density, similarly to the *arabidillo1/2* mutant (Fig. 8e). This result corroborates our finding that ARABIDILLOs do not degrade *At*MYB93, and suggests instead that an ARABIDILLO-mediated promotion of LR development in wild-type plants can be repressed by *At*MYB93 interacting with ARABIDILLOs. Thus, *At*MYB93 may integrate hormonal responses to modulate ARABIDILLO-mediated LR promotion.

## Discussion

### *At*MYB93 functions to inhibit LR development in Arabidopsis

*At*MYB93 is expressed transiently at sites of early LR development, specifically in the endodermal cells overlying developing LRPs, and is also induced by auxin in the basal meristem of the PR (a region where the patterning and spacing of LR initiation sites are regulated). *AtMYB93* loss of function is sufficient to cause an increase in the rate of LRP developmental progression and thus an enhanced LR density, while overexpression of *At*MYB93 decreases LR progression and subsequent emerged LR density. These data therefore suggest that *At*MYB93 is an early-induced inhibitor of LR development.

The enhanced LR density of the *Atmyb93* mutant is quite subtle and contrasts with the zero or severely reduced LR phenotypes seen in other well-characterized LR mutants affected in the early stages of LR development (such as *iaa14*/*slr1*, *arf7/arf19*, *iaa28* and *gata23* (Rogg *et al*., 2001; Fukaki *et al*., 2005; Okushima *et al*., 2005; De Rybel *et al*., 2010; reviewed in Peret *et al*., 2009a). This suggests a modulatory role for *At*MYB93 in LR development. We propose that the LR initiation process includes the early induction of an *At*MYB93-dependent negative feedback module capable of repressing LR development under certain conditions (e.g. stress, or changes in the nutrient status of the plant), and which is necessary for normal LR development. Future genetic analyses will allow the functional relationship between *AtMYB93* and known key positive regulators of LR initiation to be established.

The observation that the *AtMYB93* promoter is active in the endodermal cells overlying developing LRPs, rather than in the primordia themselves, implies that the overlying tissues contribute to *At*MYB93-mediated negative feedback. This cell-type-specific localization also corroborates the findings of previous studies showing that *AtMYB93* is a target of the endodermal transcription factor SCARECROW (SCR) (Iyer-Pascuzzi *et al*., 2011) and that *AtMYB93* mRNAs are significantly up-regulated in the endodermis-specific translatome (Mustroph *et al*., 2009). It was recently shown that the endodermis regulates LR initiation: PIN3 (PIN-FORMED3)-dependent auxin movement between the endodermis and the pericycle acts as a ‘checkpoint’ for initiation (Marhavy *et al*., 2013), while endodermal cells change morphology very early during the LR developmental process to accommodate pericycle cell expansion and division (Vermeer *et al*., 2014). It will be interesting to determine whether *At*MYB93 activity is linked to either of these processes. The *At*MYB93 protein may be active in the endodermis, perhaps sending a signal to the pericycle cells as LR initiation commences, or contributing to the regulation of the remodelling or separation of overlying tissues that is required for very early LRP progression (Peret *et al*., 2009b; Vermeer *et al*., 2014). Alternatively, the *At*MYB93 protein may act cell nonautonomously, moving into the pericycle or early LRPs, a phenomenon observed for other key regulatory transcription factors (Nakajima *et al*., 2001; Schlereth *et al*., 2010). Future analysis of *At*MYB93 protein localization in relation to its activity will address all of these possibilities and provide mechanistic insight into *At*MYB93 function during LR development.

### Interaction of *AtMYB93* with auxin and ABA signalling

In addition to stimulation by root bending, *AtMYB93* expression is up-regulated by auxin and ABA, two key phytohormones that regulate LR development in a complex manner (De Smet *et al*., 2003; Liang *et al*., 2007; Peret *et al*., 2009a,b; Ivanchenko *et al*., 2010). Auxin-induced up-regulation of *pAtMYB93::GUS* occurs specifically in the root basal meristem, a region where oscillating auxin sensitivity and a recurrent auxin signal determine the future position and spacing of LRs originating from the pericycle (De Smet *et al*., 2007; De Rybel *et al*., 2010; Moreno-Risueno *et al*., 2010). This suggests that *At*MYB93 may have a very early auxin-related function during LR initiation. The presence of an auxin-induced LR repressor such as *At*MYB93 in the basal meristem might help to ensure the regularity and robustness of auxin oscillation. De Smet *et al*. (2007) postulated the existence of an additional auxin response module(s) required for LR development, including an attenuation signal that ensures that LR initiation only occurs at one xylem pole at a time. It is tempting to speculate that *At*MYB93 may be involved in this process: no other candidates for an attenuation signal have been proposed to date. It is interesting to note that *AtMYB93* up-regulation by auxin is blocked in *arf7* and *arf7/19* mutants (Okushima *et al*., 2005), suggesting that *At*MYB93 functions downstream of (and could indeed be induced by) the first auxin signalling module in the basal meristem that controls LR initiation (De Rybel *et al*., 2010).

Our data suggest that *At*MYB93 is required for normal auxin signalling specifically during LR development, as the insensitivity of the *Atmyb93* mutant to higher concentrations of exogenous auxin is only observed in LRs and not in the PR. This identifies *At*MYB93 as the first known auxin-induced negative regulator specifically involved in very early LR development. The only previously identified auxin-induced negative regulator of LR development is the pleiotropic-functioning auxin signalling protein SHY2 (SHORT HYPOCOTYL2); however, SHY2 only inhibits the later stages of LR development and is in fact a positive regulator of initiation events (Tian & Reed, 1999; Swarup *et al*., 2008; Goh *et al*., 2012). It may seem surprising that *Atmyb93* mutants are insensitive to higher concentrations of exogenously applied auxin, given that *AtMYB93* is an auxin-induced negative regulator of LRs. However, we suggest that if normal LR development requires a functional negative feedback loop, then loss of the feedback loop may block further auxin-induced LR development. The fact that *Atmyb93* mutants are neither insensitive nor hypersensitive to concentrations of exogenous auxin below 1 μM confirms that additional *At*MYB93-independent feedback mechanisms (for example, a SHY2 module) are also likely to regulate normal LR development.

In addition to auxin, *AtMYB93* gene expression is induced by exogenous ABA. *Atmyb93* mutant LRs respond to ABA similarly to wild-type and no additional induction of *pAtMYB93::GUS* by ABA is seen in the seedling root, suggesting that ABA does not play a role in the control of LR development by *At*MYB93. We cannot rule out the possibility that ABA plays an additional regulatory role(s) under certain conditions or stresses, or at other stages in the plant life cycle. Interestingly, *AtMYB93* is a direct transcriptional target of SCR, which defines the cell identity of the root endodermis and cortex, regulates ABA-mediated germination and is differentially regulated in the root by various abiotic stresses (Iyer-Pascuzzi *et al*., 2011). Moreover, the endodermis regulates later LR developmental progression in response to salt stress (Duan *et al*., 2013), and LR responses to environmental stress differ from PR responses (Tian *et al*., 2014), suggesting that *At*MYB93, which has an LR-specific function, could also have stress-responsive roles. This will be addressed in future studies.

### *AtMYB93* is part of a small gene family in Arabidopsis with relatives in other flowering plants

*AtMYB93* is one of three related Arabidopsis ARABIDILLO1-interacting R2R3 MYB genes and is the only member of its subclade with root-restricted expression. We have shown that *At*MYB93 is also the only auxin-responsive member of its subclade, corroborating the findings of previous large-scale and LR-specific transcriptional studies (this work; Kranz *et al*., 1998; Okushima *et al*., 2005; Yanhui *et al*., 2006; Winter *et al*., 2007; Lewis *et al*., 2013). As *At*MYB93 is divergent from *At*MYB92 and *At*MYB53, we suggest that *AtMYB93* may have acquired novel root-regulating functions during evolution. *At*MYB93 functions only partially redundantly with its relative(s), as the *Atmyb92* mutant has no LR phenotype. *AtMYB92* and *AtMYB53* are expressed throughout the plant: future studies will address the functions of these genes during plant development.

Homologues of *AtMYB93* and its relatives are present in flowering plants but appear to be absent from plants lacking LRs, namely bryophytes and lycophytes. This is in contrast to ARABIDILLO proteins, which are very highly conserved across all land plants (Nibau *et al*., 2011; Moody *et al*., 2012). It seems likely that ARABIDILLO proteins evolved an LR-promoting function either after the divergence of the flowering plant lineage or after the evolution of true LRs. Identification of *At*MYB93 homologues in gymnosperms and ferns would help to resolve these scenarios. We cannot currently distinguish between the possibilities that (i) early-evolving ARABIDILLO proteins could interact with more divergent MYBs in early land plants and perform nonroot functions and (ii) early-evolving ARABIDILLOs could have non-MYB interaction partners.

### How does *At*MYB93 functionally interact with ARABIDILLO proteins?

*At*MYB93 and its close homologues in Arabidopsis interact with ARABIDILLO1, a positive regulator of LR initiation (Coates *et al*., 2006). Given that ARABIDILLO proteins are F-box proteins that associate with the SKP1 (S-phase kinase-associated protein 1) component of the proteasomal degradation machinery, our previous hypothesis was that ARABIDILLO proteins target an inhibitor of LR formation, such as *At*MYB93, for degradation (Nibau *et al*., 2011). However, our experiments have not detected any regulation of *At*MYB93 stability by ARABIDILLO proteins. Moreover, we previously showed that mutating key F-box residues in ARABIDILLO1 does not abolish the protein's *in planta* function during LR formation, indicating that its function during the promotion of LR development is not linked to its putative role as an E3 ligase (Nibau *et al*., 2011). Thus, an alternative possibility is that ARABIDILLO and MYB proteins could interact to form a functional protein complex that regulates the transcription of downstream genes, with ARABIDILLOs promoting LR development and *At*MYB93 binding as a repressor. This scenario is similar to what is seen with animal beta-catenin/Armadillo proteins (to which ARABIDILLOs are structurally related), which interact with both transcriptional activators and repressors (Valenta *et al*., 2012). The *arabidillo*-like phenotype of the *arabidillo1/arabidillo2/Atmyb93* triple mutant supports this possibility. We suggest that, in the absence of *At*MYB93 (the *Atmyb93* single mutant), ARABIDILLOs can promote LR development to a greater extent than in wild-type plants, whereas in the absence of ARABIDILLO proteins the promotion of LR development can no longer occur, regardless of the presence of *At*MYB93. The functional mechanism of the interaction between *At*MYB93 and ARABIDILLO will be the target of future study. Furthermore, future focus will be placed on identifying ARABIDILLO and *At*MYB93 gene targets and uncovering how these modifiers of LR development interact with the myriad other signalling components that regulate this complex and highly plastic developmental process.

**About New Phytologist**

- *New Phytologist* is an electronic (online-only) journal owned by the New Phytologist Trust, a **not-for-profit organization** dedicated to the promotion of plant science, facilitating projects from symposia to free access for our Tansley reviews.
- Regular papers, Letters, Research reviews, Rapid reports and both Modelling/Theory and Methods papers are encouraged. We are committed to rapid processing, from online submission through to publication ‘as ready’ via *Early View* - our average time to decision is <25 days. There are **no page or colour charges** and a PDF version will be provided for each article.
- The journal is available online at Wiley Online Library. Visit http://www.newphytologist.com to search the articles and register for table of contents email alerts.
- If you have any questions, do get in touch with Central Office (np-centraloffice@lancaster.ac.uk) or, if it is more convenient, our USA Office (np-usaoffice@ornl.gov)
- For submission instructions, subscription and all the latest information visit **http://www.newphytologist.com**

## Supporting Information

**Fig. S1** Protein sequence alignment of *At*MYB93, -92 and -53 homologues in land plants. **Fig. S2** A second *Atmyb93* allele, *Atmyb93-2*, shows increased LR formation. **Fig. S3** An *Atmyb92* single mutant has no PR or LR phenotypes. **Fig. S4** PR length is slightly reduced relative to wild-type in *35S::MYC-AtMYB93* seedlings. **Fig. S5** qRT-PCR analysis of the effect of exogenous IAA and ABA on *MYB92* and *MYB53* expression. **Fig. S6** *Atmyb93* mutants grown on NPA for 7 d and then transferred to 0.5× MS are still able to produce more lateral roots than wild-type plants. **Fig. S7** qRT-PCR analysis of *ARABIDILLO1* and *AtMYB* genes in *arabidillo1/2*, *Atmyb93* and *Atmyb92/Atmyb93* mutants.

**Table S1** List of primers used in this work
