# Supplementary material for: AtMYB93 is a novel negative regulator of lateral root development in Arabidopsis
Source: New Phytol. 2014 Jun 6;203(4):1194–207. doi: 10.1111/nph.12879 (PMC4286813; doi:10.1111/nph.12879)
Supplement: Supplementary file 1 [file nph0203-1194-SD1.pptx]

## Slide 1
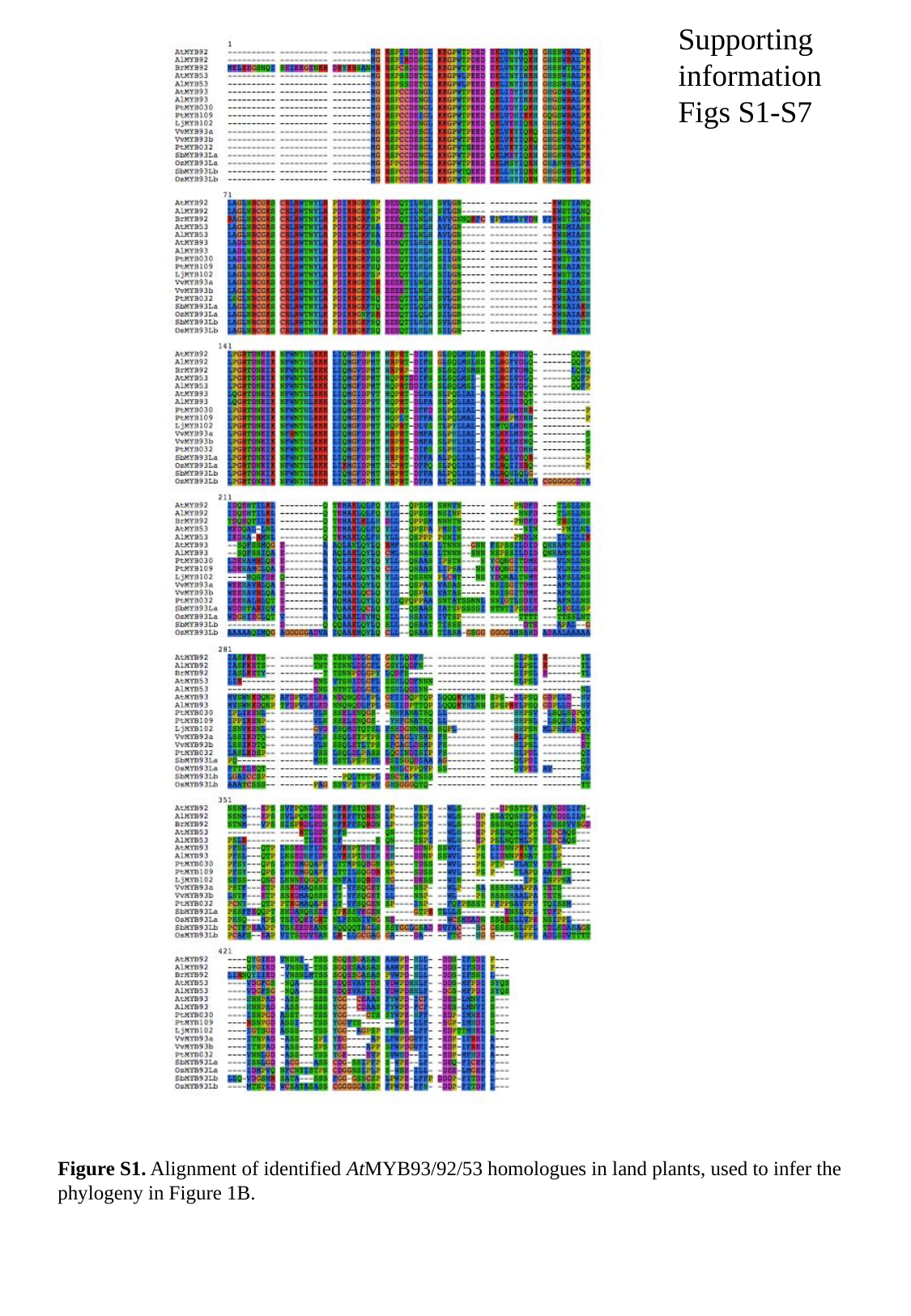

Supporting information
Figs S1-S7
Figure S1. Alignment of identified AtMYB93/92/53 homologues in land plants, used to infer the phylogeny in Figure 1B.

## Slide 2
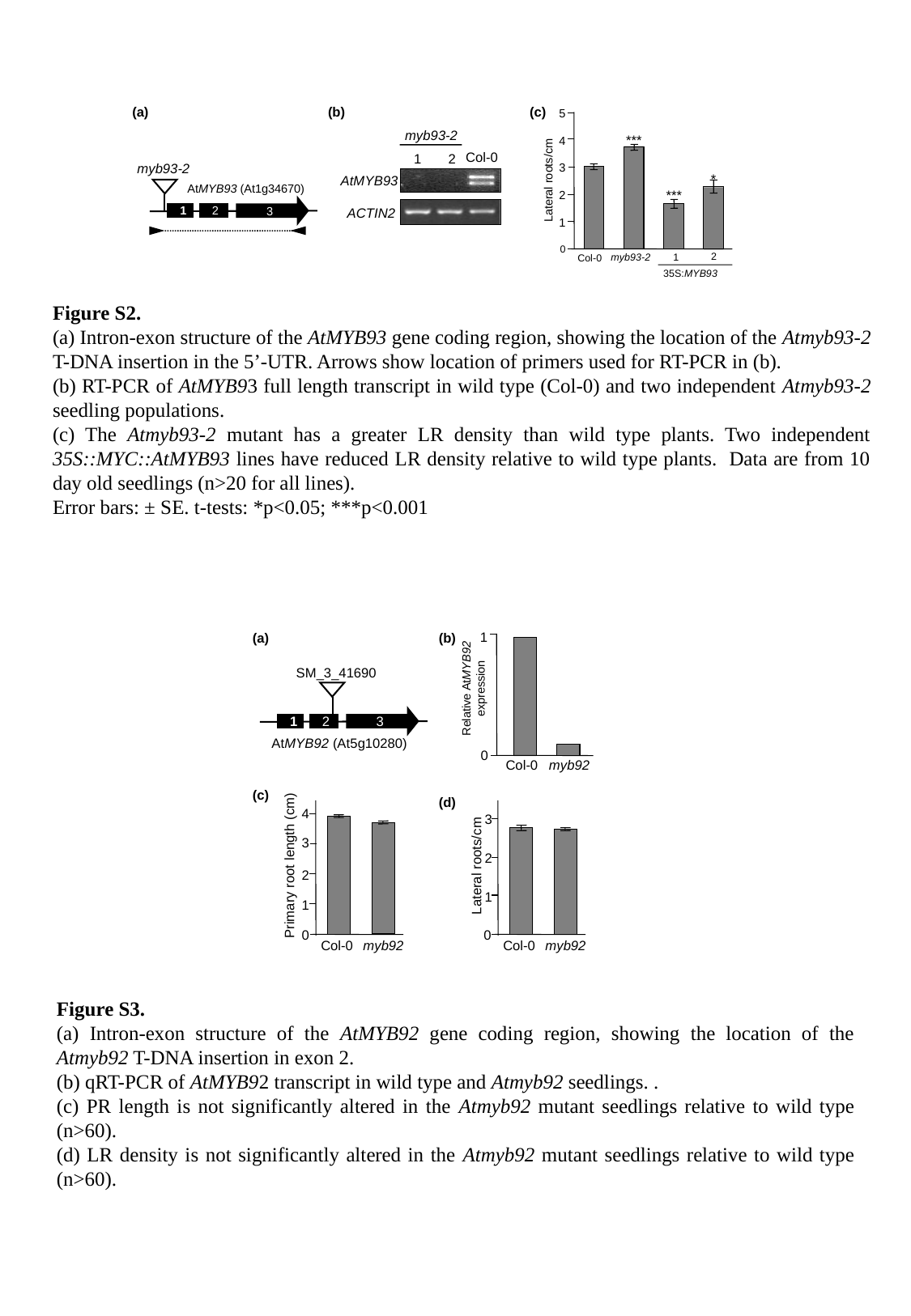

(a)
(b)
(c)
5
myb93-2
Col-0
 1 2
AtMYB93
ACTIN2
***
4
myb93-2
3
*
Lateral roots/cm
AtMYB93 (At1g34670)
***
2
3
1
2
1
0
2
myb93-2
1
Col-0
35S:MYB93
Figure S2.
(a) Intron-exon structure of the AtMYB93 gene coding region, showing the location of the Atmyb93-2 T-DNA insertion in the 5’-UTR. Arrows show location of primers used for RT-PCR in (b).
(b) RT-PCR of AtMYB93 full length transcript in wild type (Col-0) and two independent Atmyb93-2 seedling populations.
(c) The Atmyb93-2 mutant has a greater LR density than wild type plants. Two independent 35S::MYC::AtMYB93 lines have reduced LR density relative to wild type plants. Data are from 10 day old seedlings (n>20 for all lines).
Error bars: ± SE. t-tests: *p<0.05; ***p<0.001
1
(a)
(b)
 SM_3_41690
3
1
2
AtMYB92 (At5g10280)
Relative AtMYB92 expression
0
Col-0
 myb92
(c)
(d)
4
3
3
2
Lateral roots/cm
Primary root length (cm)
2
1
1
0
0
Col-0
myb92
Col-0
myb92
Figure S3.
(a) Intron-exon structure of the AtMYB92 gene coding region, showing the location of the Atmyb92 T-DNA insertion in exon 2.
(b) qRT-PCR of AtMYB92 transcript in wild type and Atmyb92 seedlings. .
(c) PR length is not significantly altered in the Atmyb92 mutant seedlings relative to wild type (n>60).
(d) LR density is not significantly altered in the Atmyb92 mutant seedlings relative to wild type (n>60).

## Slide 3
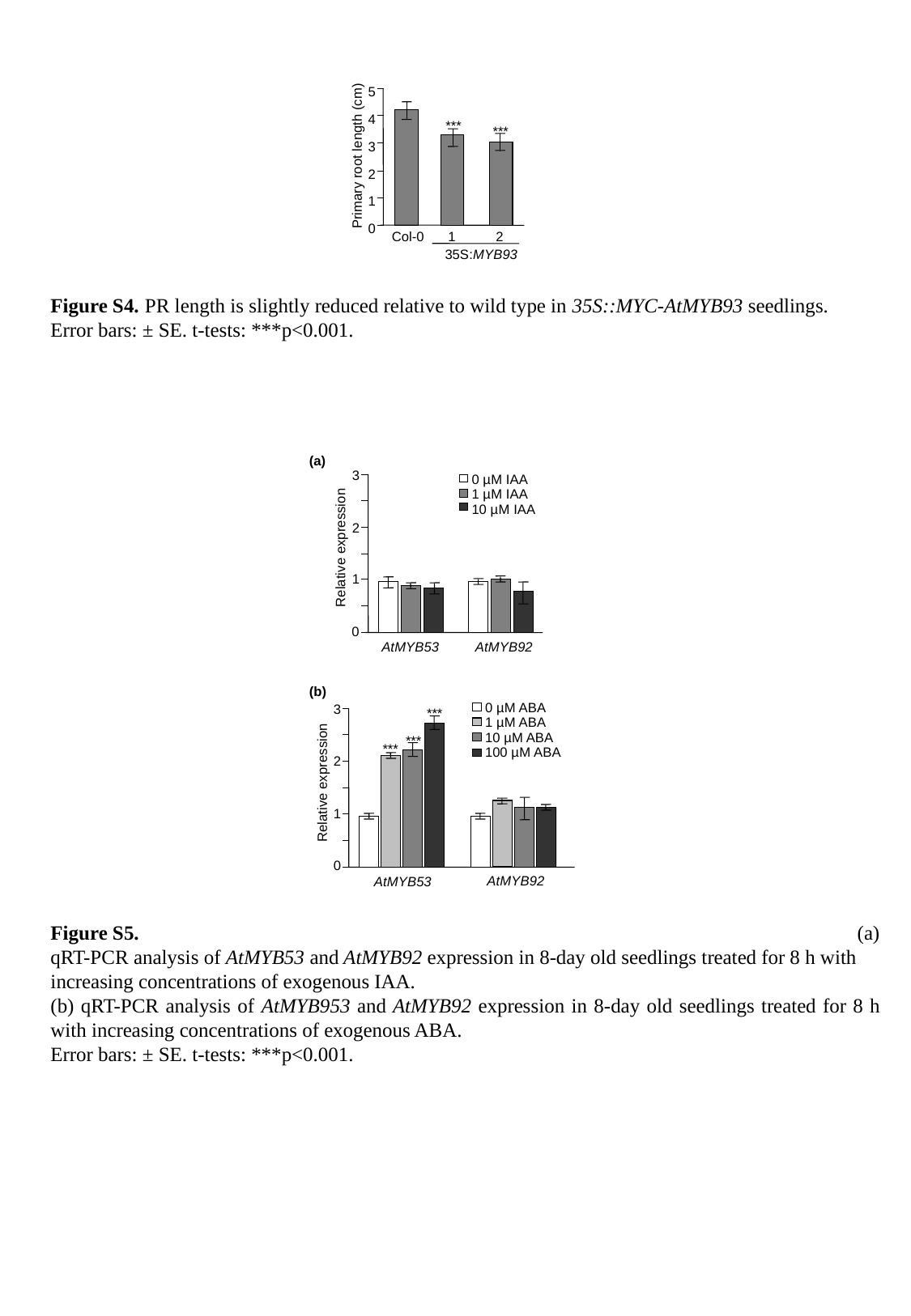

5
4
***
***
3
Primary root length (cm)
2
1
0
Col-0
1
2
35S:MYB93
Figure S4. PR length is slightly reduced relative to wild type in 35S::MYC-AtMYB93 seedlings.
Error bars: ± SE. t-tests: ***p<0.001.
(a)
3
0 µM IAA
1 µM IAA
10 µM IAA
2
Relative expression
1
0
AtMYB53
AtMYB92
(b)
3
***
0 µM ABA
1 µM ABA
10 µM ABA
100 µM ABA
***
***
2
Relative expression
1
0
AtMYB92
AtMYB53
Figure S5. (a) qRT-PCR analysis of AtMYB53 and AtMYB92 expression in 8-day old seedlings treated for 8 h with increasing concentrations of exogenous IAA.
(b) qRT-PCR analysis of AtMYB953 and AtMYB92 expression in 8-day old seedlings treated for 8 h with increasing concentrations of exogenous ABA.
Error bars: ± SE. t-tests: ***p<0.001.

## Slide 4
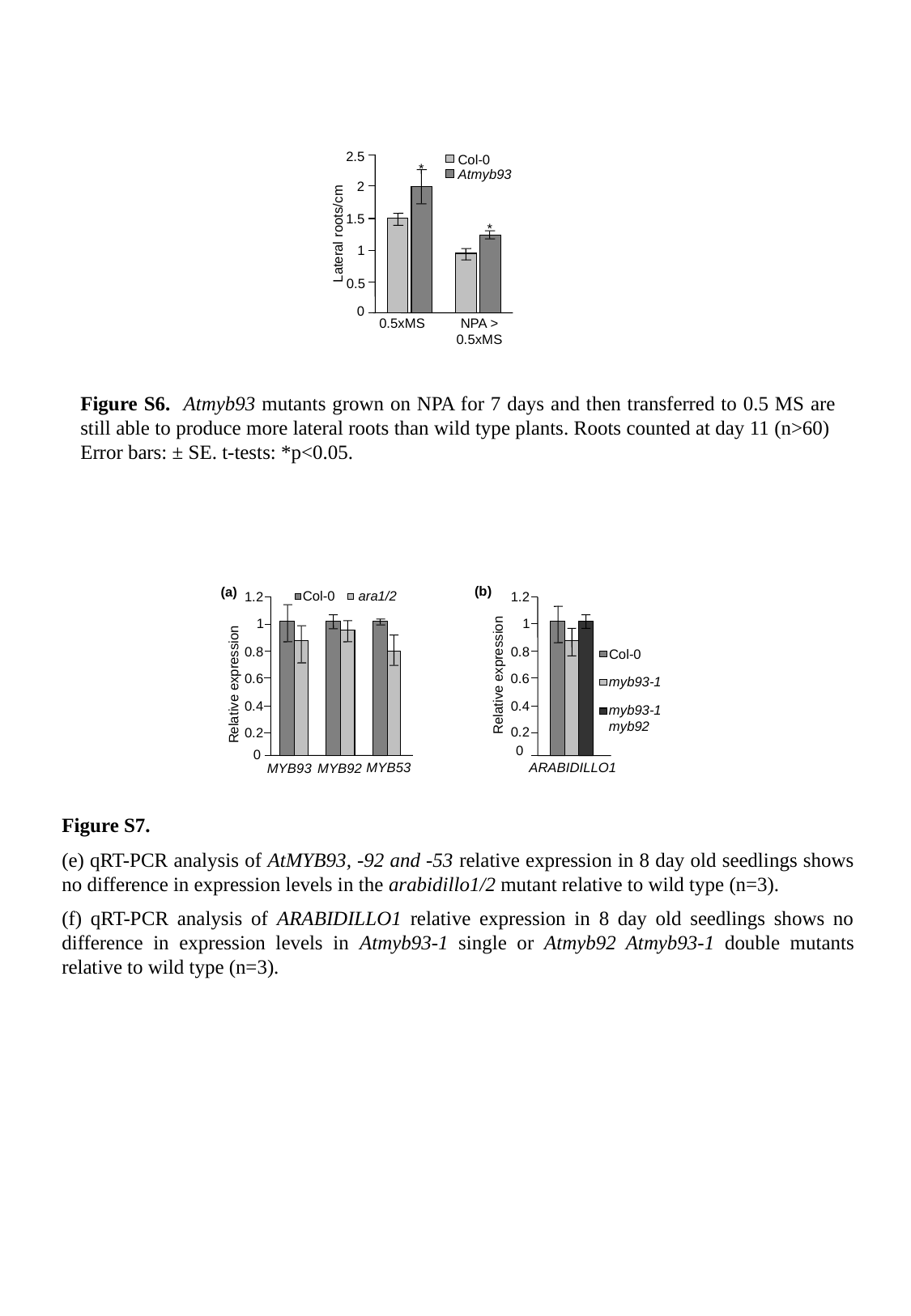

2.5
Col-0
Atmyb93
*
2
1.5
*
Lateral roots/cm
1
0.5
0
0.5xMS
NPA > 0.5xMS
Figure S6. Atmyb93 mutants grown on NPA for 7 days and then transferred to 0.5 MS are still able to produce more lateral roots than wild type plants. Roots counted at day 11 (n>60)
Error bars: ± SE. t-tests: *p<0.05.
(b)
(a)
Col-0
ara1/2
1.2
1.2
1
1
0.8
0.8
Col-0
myb93-1
myb93-1 myb92
Relative expression
0.6
0.6
Relative expression
0.4
0.4
0.2
0.2
0
0
MYB53
ARABIDILLO1
MYB93
MYB92
Figure S7.
(e) qRT-PCR analysis of AtMYB93, -92 and -53 relative expression in 8 day old seedlings shows no difference in expression levels in the arabidillo1/2 mutant relative to wild type (n=3).
(f) qRT-PCR analysis of ARABIDILLO1 relative expression in 8 day old seedlings shows no difference in expression levels in Atmyb93-1 single or Atmyb92 Atmyb93-1 double mutants relative to wild type (n=3).
